# Editorial: Environmental, Clinical, and Biological Determinants of Preterm Birth and Their Effects on the Offspring

**DOI:** 10.3389/frph.2021.795962

**Published:** 2021-11-12

**Authors:** Manuel L. Wolfson, Stefania I. Papatheodorou, Fernando Correa

**Affiliations:** ^1^Centro de Estudios Farmacológicos y Botánicos, Universidad de Buenos Aires-Consejo Nacional de Investigaciones en Ciencia y Técnica, Ciudad Autónoma de Buenos Aires, Buenos Aires, Argentina; ^2^School of Public Health, Harvard University, Boston, MA, United States

**Keywords:** preterm (birth), biological factors, environmental factors, clinical factors, offspring

This editorial aims to summarize the 5 scientific papers that contributed to the Research Topic: “*Environmental, Clinical, and Biological Determinants of Preterm Birth and their Effects on the Offspring*.” The main goal of this special collection was to bring together the most recent evidence on modifiable risk factors for preterm birth (PTB) in order to inform clinicians, public health officials and patients on current advances on PTB research and propose effective interventions.

Preterm birth is defined as birth occurring before gestational week 37, and represents the leading cause of child mortality in children under 5 years old. Around 15 million babies are born prematurely worldwide each year, namely more than one in ten births. Despite the advances in pre- and periconceptional care as well as advances to neonatal care, these numbers remain quite stable. Although the etiology of PTB remains elusive, it is well-known that social, cultural, environmental, clinical, and genetic factors contribute to the pathogenesis of this event.

Here, Xu et al. performed a large-scale population-based retrospective cohort study including over 4.9 million Chinese women. These authors found that the association between PBT and body mass index (BMI) varied depending on the fasting plasma glucose (FPG) levels. Consequently, Xu et al. showed that the shapes of preconception BMI and the risk of PTB were generally U-shaped in women with normal glucose or prediabetes, and J-shaped in women with diabetes, with the risk of PTB increasing with the increase of BMI. Interestingly, the curve in women with hypoglycemia was approximately L-shaped. For the association of FPG with PTB; the dose-response relationships were U-shaped in women with normal weight, overweight, and obesity, and L-shaped in underweight women. Given that the association between BMI and PTB has been considered to be U-shaped, this study provides insight on the differences that may exist in different subgroups of the population, dictating different appropriate preventive measures.

On the other hand, Acharya et al. identified risk factors for PTB in Nepal, which holds the 20th position globally for preterm deliveries. This low-income South Asian nation still has high neonatal mortality rate, despite the improvements in the recent years. The authors of this study found that Nepalese women who did not receive anthelminthic treatment had higher odds for PTB than women who had anthelminthic treatment. Likewise, women who were expose to indoor air pollution or preformed intensive work during pregnancy were more susceptible to experience PTB. The results of this study clearly demonstrate that simple and inexpensive interventions should be considered in certain countries at the population level and reductions in ambient air pollution can also have a greater margin of improvement in the prevalence of PTB.

Jain et al. performed a comprehensive systematic review of environmental stressors and climate change on maternal-fetal health in India. They also evaluated the role of the international program Optimal Pregnancy Environment Risk Assessment (OPERA), supported by the Worldwide Universities Network, World Health Organization and March of Dimes USA aiming at helping local practitioners by sharing with them the latest risk prediction tools in pregnancy for early identification of high risk populations, as well as the easy and inexpensive interventions to mitigate those risks.

In their article, Megaw et al. address the question whether an environmental factor such as a meteorological condition could impact the incidence of PTB. The authors found that higher first trimester sunlight exposure is associated with a lower PTB risk in Scotland. Similar patterns were seen on sibling analysis and within both the indicated and spontaneous preterm subgroups. This study truly reveals new mechanisms, and potential therapeutic pathways, for preterm birth prevention.

Finally, Braveman et al. from a multi-disciplinary scientific work group convened by the March of Dimes, reviewed the literature on the different hypotheses on the Black-White disparity in PTB, including biomedical, clinical, epidemiologic, and social factors. The authors divide the potential causes into *downstream causes* (immediate causes such as infection, nutrition, and hypertensive diseases of pregnancy that directly trigger the biological mechanisms producing PTB), *midstream causes* (such as socioeconomic factors and stress, which influence the downstream factors) and a single *upstream cause*, racism. Even though the authors propose a thought-provoking hypothesis, it seems to be rather US-centric where it seems quite plausible that part of the burden of the disparities in Black-White in PTB rate can be attributed to racism.

Overall, these 5 contributions published in this Research Topic provide valuable information on the different factors influencing PBT in China (Xu et al.), Nepal (Acharya et al., and Scotland) (Megaw et al.), as well as an ongoing program in India aimed at helping local health professional to better manage risks during pregnancy (Jain et al.), and how racism could function as a master regulator of midstream and downstream causes of PTB in the USA. Given the representation of broad geographic areas, this collection of articles provides both an in-depth assessment of the distinct characteristics of each country's environment while providing a global perspective on the issues future research needs to focus on to reduce the burden of PTB and improve the short- and long-term health of the mother and the fetus.

## Author Contributions

All authors listed have made a substantial, direct and intellectual contribution to the work, and approved it for publication.

## Funding

MW was supported by Agencia Nacional para la Promoción Científica y Tecnológica (ANPCyT PICT 2018-1489 and PICT 2019-1786) del Ministerio de Ciencia y Tecnología (Argentina). FC was also supported by Agencia Nacional para la Promoción Científica y Tecnológica (ANPCyT PICT 2016-0129 and PICT 2016-0803) del Ministerio de Ciencia y Tecnología (Argentina).

## Conflict of Interest

The authors declare that the research was conducted in the absence of any commercial or financial relationships that could be construed as a potential conflict of interest.

## Publisher's Note

All claims expressed in this article are solely those of the authors and do not necessarily represent those of their affiliated organizations, or those of the publisher, the editors and the reviewers. Any product that may be evaluated in this article, or claim that may be made by its manufacturer, is not guaranteed or endorsed by the publisher.

